# Endocrine Tumor Classification via Machine-Learning-Based Elastography: A Systematic Scoping Review

**DOI:** 10.3390/cancers15030837

**Published:** 2023-01-29

**Authors:** Ye-Jiao Mao, Li-Wen Zha, Andy Yiu-Chau Tam, Hyo-Jung Lim, Alyssa Ka-Yan Cheung, Ying-Qi Zhang, Ming Ni, James Chung-Wai Cheung, Duo Wai-Chi Wong

**Affiliations:** 1 Department of Biomedical Engineering, Faculty of Engineering, The Hong Kong Polytechnic University, Hong Kong, China; 2 Department of Bioengineering, Imperial College London, London SW7 2AZ, UK; 3 Department of Electronic Engineering, Faculty of Engineering, The Chinese University of Hong Kong, Hong Kong, China; 4 Department of Orthopaedics, Tongji Hospital Affiliated to Tongji University, Shanghai 200065, China; 5 Department of Orthopaedics, Shanghai Pudong New Area People’s Hospital, Shanghai 201299, China; 6 Department of Orthopaedics, Ruijin Hospital, Shanghai Jiao Tong University, Shanghai 200025, China; 7 Research Institute of Smart Ageing, The Hong Kong Polytechnic University, Hong Kong, China

**Keywords:** neoplasia, neoplasm, cancer, neuroendocrine tumor, computer-aided diagnosis, deep learning, artificial intelligence, sonoelastography

## Abstract

**Simple Summary:**

The incidence of endocrine cancers (e.g., thyroid, pancreas, and adrenal) has been increasing; these cancers have a high premature mortality rate. Traditional medical imaging methods (e.g., MRI and CT) might not be sufficient for accurate cancer screening. Elastography complements conventional medical imaging modalities by identifying abnormal tissue stiffness of the tumor, in which machine learning techniques can further improve accuracy and reliability. This review focuses on the applications and performance of machine-learning-based elastography in classifying endocrine tumors.

**Abstract:**

Elastography complements traditional medical imaging modalities by mapping tissue stiffness to identify tumors in the endocrine system, and machine learning models can further improve diagnostic accuracy and reliability. Our objective in this review was to summarize the applications and performance of machine-learning-based elastography on the classification of endocrine tumors. Two authors independently searched electronic databases, including PubMed, Scopus, Web of Science, IEEEXpress, CINAHL, and EMBASE. Eleven (*n* = 11) articles were eligible for the review, of which eight (*n* = 8) focused on thyroid tumors and three (*n* = 3) considered pancreatic tumors. In all thyroid studies, the researchers used shear-wave ultrasound elastography, whereas the pancreas researchers applied strain elastography with endoscopy. Traditional machine learning approaches or the deep feature extractors were used to extract the predetermined features, followed by classifiers. The applied deep learning approaches included the convolutional neural network (CNN) and multilayer perceptron (MLP). Some researchers considered the mixed or sequential training of B-mode and elastographic ultrasound data or fusing data from different image segmentation techniques in machine learning models. All reviewed methods achieved an accuracy of ≥80%, but only three were ≥90% accurate. The most accurate thyroid classification (94.70%) was achieved by applying sequential training CNN; the most accurate pancreas classification (98.26%) was achieved using a CNN–long short-term memory (LSTM) model integrating elastography with B-mode and Doppler images.

## 1. Introduction

The endocrine system plays an essential role in regulating metabolism by synthesizing and releasing hormones into the body and transporting hormones to target cells [1]. The cellular processes of the target cells are directly or indirectly modulated when the hormones bind to the receptor molecules [1]. In addition, the endocrine system works together with other systems to maintain normal physiological activities of the human body. The abnormal growth of nodules or tumors in the endocrine system affects normal hormone production and can result in various diseases [2]. The global burden of endocrine-related cancers is increasing because of aging and exposure to alcohol, high-fat diets, and tobacco [3]. For example, thyroid cancer has been the most common endocrine malignancy over the past few decades, accounting for approximately 2% of all cancers [4,5]. Additionally, pancreatic cancer has a poor prognosis, with a 5-year survival rate of approximately 2.5% [6]. The incidence of the most common endocrine tumor, thyroid cancer, has dramatically increased in the United States, with approximately 53,990 cases [7]. Additionally, more than half of the new tumor cases in China are metastatic [8]. The initial diagnosis and treatment of thyroid cancers cost USD 1425 to 17,000 [9]. A total of 9.3 per 1000 person-years of patients experience financial catastrophe one year postdiagnosis, which is a substantially higher rate than for other cancers [9]. Survivors bear additional psychological and financial burdens and experience monetary hardship [10,11]. Approaching the highest mortality rate among all cancers, the global burden of pancreatic cancer has doubled in the past decades [12,13]. Early and accurate diagnosis of pancreatic cancer can facilitate more efficacious treatments [14], whereas identifying thyroid cancer at earlier stages can improve prognosis and reduce patient mortality [15].

Medical imaging modalities, such as magnetic resonance imaging (MRI), computed tomography (CT), and ultrasonography, are vital assessment and diagnostic tools. Ultrasonography is mainly used to screen and evaluate the characteristics of thyroid nodules [16,17], and real-time ultrasonography supports other assessment modalities, such as fine-need aspiration, biopsy, cytology, etc. [17,18]. Additionally, magnetic resonance imaging (MRI) is the mainstay imaging modality in staging pancreatic cancer [19,20] and evaluating the neuroanatomy for pituitary adenomas [21,22]. Locating and measuring the size of adrenal tumors is more challenging and requires the use of contrast-enhanced computed tomography (CT) [16].

These medical imaging modalities have several drawbacks. CT exposes patients to radiation and may not be appropriate for the frequency tracking of tumor progression [23]. Functional MRI is susceptible to noise and articles and has insufficient temporal and spatial resolution and a low signal-to-noise ratio [24] and is contraindicated for patients with metallic implants or pacemakers. Additionally, ultrasound might be limited by the penetration depth and spatial resolution [25]. Therefore, an accurate and accessible tumor assessment imaging modality that reduces radiation risk is required.

Elastography is an emerging imaging technology that measures and maps tissue stiffness/elasticity, inspired by the manual palpation technique [26]. Despite sharing the same limitations in terms of penetration depth and spatial resolution as B-mode ultrasound, it provides complementary mechanical information of tissues. With this method, tissue abnormality (malignancy) affects its ability to resist load deformation (i.e., stiffness) [27]. Ultrasound elastography has been widely used in different medical applications, such as for the spine [28,29], breast [30], liver [31], brain [32,33,34], and lymph nodules [35]. The two types of elastography are shear-wave and strain imaging. In the strain imaging technique, a force is applied to the tissue and the strain is measured for calculating Young’s modulus. In contrast, in shear-wave imaging, tissue stiffness is estimated by measuring the propagation velocity of shear waves [27]. Furthermore, MRI elastography has also been adopted for elastography in the assessment of chronic disease, such as lung disease [36], hepatic fibrosis [37], breast cancer [38], etc.

Recently, computer-aided diagnosis was found to improve the diagnostic performance and reliability of medical imaging [39]; this method is also less operator-dependent and less prone to observer variability [40,41,42]. Machine learning (and deep learning) models play an important role in computer-aided diagnosis. Using mathematics and statistics tools, machine learning models extract and segment relevant features, interpret the output, and formulate a predictive model by correlating the data with the diagnosis of the patients [43]. For example, machine learning was applied to contrast-enhanced CT to distinguish large adrenocortical carcinomas from other cortical lesions [44]. However, in addition to requiring a large dataset, some models may not have sufficient power to produce a satisfactory performance in the image segmentation of a specific modality [45]. With the advancement of machine learning techniques, especially deep learning models, we anticipate that the technique will also be applied in elastography for endocrine tumor classification [46].

The aim of this study was to provide a contemporary and comprehensive literature review on the application of machine-learning-based elastography to classify endocrine tumors, including thyroid, pancreas, adrenal, and pituitary tumors.

## 2. Materials and Methods

### 2.1. Search Strategy

In our systematic literature search, we followed the guidance of the Preferred Reporting Items for Systematic Review and Meta-Analysis Protocols Extension for Scoping Reviews (PRISMA-ScR) guidelines [47], which we conducted on: PubMed (title/abstract, journal articles, English), SCOPUS (title/abstract/keywords), Web of Science (topic field, articles, English), IEEEXpress (title/abstract/indexing terms), CINAHL via EBSCOhost (title/abstract/keywords), and EMBASE via OVID (title/abstract/author keywords, English).

Two authors (Y.-J.M. and L.-W.Z.) conducted independent searches in August 2022. The first author (Y.-J.M.) screened abstracts and full texts, which were checked by another author (L.-W.Z.) Any disagreement was resolved by seeking consensus with the corresponding authors.

We searched the literature with a combination of keywords related to the areas of endocrine tumors, elastography, and machine learning. For endocrine tumors, the searching terms were “thyroid”, “pancrea*”, “adrenal”, or “endocrine” and those with “nodule*”, “tumo*r*”, “cancer”, “carcinoma*”, “malignan*”, “neoplas*”, or “mass*”. For elastography, the search terms were “elastograph*”, or “sonoelastograph*”. For machine learning, the searching terms were “machine learning”, “deep learning”, “neural network”, “CNN”, “RNN”, “ANN”, and “cascade network*”. The raw search and operations are included in Table S1.

The search was limited to original journal research articles published in English. The inclusion criteria included (1) application elastography (in any modality) to classify endocrine tumors; (2) deep learning or machine learning technique involving image segmentation, feature extraction, and classification; (3) classifying benign and malignancy; (4) studies conducted on human subject or existing human subject data; and (5) with at least one classification-related performance measure. Studies were excluded if they (1) had insufficient details on the machine learning model; (2) were modeled or evaluated by purely simulated data; or (3) classified metastasis.

### 2.2. Screening and Data Extraction

**Figure 1:**
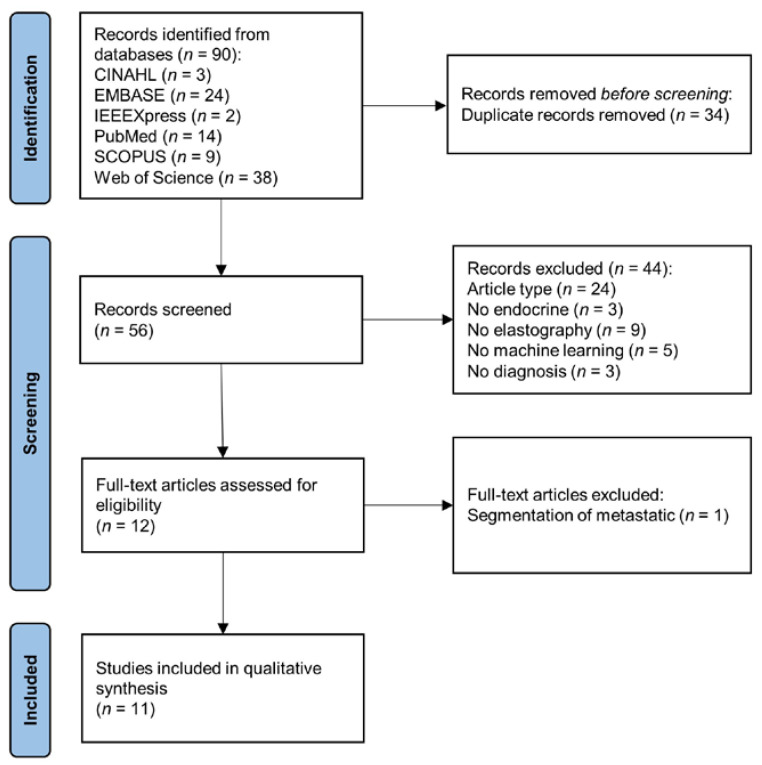
PRISMA flowchart of systematic search and screening.

The PRISMA flowchart shown in Figure 1 illustrates the search and screening process for this systematic review. The review context included the basic information (Table 1), the configuration of the elastography system, image preprocessing and segmentation (Table 2), feature extraction and classification (Table 3), evaluation metrics, and performance (Table 4).

## 3. Results

### 3.1. Search Results

As shown in Figure 1, the initial search yielded 90 articles. After the exclusion of duplicates, 56 articles remained. A preliminary screening of the title and abstract led to the removal of 44 articles, for the following reasons: article type, *n* = 24; not related to endocrine, *n* = 3; no elastography, *n* = 9; no machine learning, *n* = 5; unrelated to tumor classification, *n* = 3. One article about the segmentation of metastatic was excluded during full-text screening. In the end, 11 articles were eligible for data synthesis [48,49,50,51,52,53,54,55,56,57,58].

### 3.2. Basic Information and Dataset

The 11 articles involved a total of 5612 participants with sample sizes ranging from 65 to 2032 and patient age ranging from 15 to 90 year, as shown in Table 1. All except two studies were published in or after 2018. Though we covered thyroid, pancreas, adrenal, and pituitary tumors in the literature search, only those on the pancreas and thyroid were found, accounting for three (*n* = 3) and eight (*n* = 8) studies, respectively. One study further classified tumors into pseudotumoral pancreatitis, neuroendocrine tumor, and ductal adenocarcinoma [54].

Women were generally more prevalent in thyroid research, whereas men were more prevalent in pancreas studies. Most of the studies (8/11) confirmed the diagnosis (i.e., ground truth) by biopsy, whereas the others did not specify how they determined malignancy. Furthermore, the lesion size of the tumors was not available in five articles, which can be an important factor in image processing and classification.

As shown in Table 2, in all reviewed studies, researchers used ultrasound elastography and no researchers applied the magnetic resonance elastography. Of the 11 articles, shear-wave ultrasound elastography was used in 6, all of which targeted thyroid tumors. In the contrast, in four articles, the authors used strain elastography and all of them targeted pancreas tumors. Two studies did not provide sufficient information on the type of ultrasound elastography, and four articles did not provide the name/brand of the system.

## 4. Review Theme and Context

### 4.1. Data Processing and Segmentation

As shown in Table 2, for data processing, one study [53] mentioned the application of a median filter for denoising, whereas two studies highlighted the process of contrast enhancement of the acquired images [54,58]. However, other studies did not address any image processing or conditioning (excluding segmentation).

For data segmentation, delineating the region of interest (ROI) is one of the essential steps in image processing to focus the center of attention on the clinically relevant regions and to avoid irrelevant image area information from degrading the efficiency and accuracy of model training. The procedures were often conducted by manually contouring the tumor boundary by radiologists with the assistance of software [42,53,54,59]. Alternatively, Pereira et al. [49] applied a threshold-based method to segment the regions with elastographic stress higher than 70% of the maximum stress, but this threshold level was not justified. Qin et al. [50] pre-extracted the ROI using a color transformation technique before the manual work by the radiologists.

Elastographic images can be segmented by overlaying segmented B-mode ultrasound images. Hu et al. [48] trained a real-time semantic segmentation model, PP-LiteSeg [60], on B-mode ultrasound images for segmentation. Then, they copied the segmented outline from B-mode images to the elastographic images with different offsets. The accuracy of the PP-LiteSeg model was verified by radiologists using the dice similarity coefficient, Cohen’s kappa, and 95% symmetric Hausdorff distance.

Data augmentation was implemented by a few authors. The traditional data augmentation involves random transformation, flipping, and scaling [48,50,54]; Hu et al. [48] also considered augmentation of the brightness, contrast, and saturation of the images. In addition, some researchers considered integrating different segmentation methods or combining lower- and higher-dimensional semantic features as a form of data augmentation [48,50].

### 4.2. Feature Extraction and Data Fusion

Using predetermined statistical-based features is one of the common strategies applied in feature extraction. In addition, some researchers extracted features from B-mode ultrasound [49,53,54,55,57] and Doppler ultrasound [54] for tumor classification, as shown in Table 3.

The statistical features of elastographic images include the mean, standard deviation, range, and highest stress value [49,57]. Pereira et al. [49] also considered the number of pixels with a stress level greater than 80 kPa but without justification. In addition, they applied the circular Hough transform to obtain additional features, including the largest radius detected, the largest value of the accumulator array, and the radius corresponding to the largest value on the accumulator array [49]. Additionally, Zhou et al. [58] extracted features based on the gray-level co-occurrence matrix and gray-level run-length matrix (GLCOM-GLRLM), as well as the multiple subgraph co-occurrence matrix based on multilevel wavelet (MSCOM). GLCOM-GLRLM represented the length of the highest highlight run continuously distributed in the image, whereas MSCOM was used to mark the image area with stripe-like textures [58].

Radiomics features were also considered in these studies, which are different from statistical features in that they are generally ordinal or categorical data classified by radiologists. For example, researchers [56] identified the shape and smoothness of a nodule, the nature of the calcification, and the vascularity (in the four-grade Alder classification scheme). The authors continued by automating the radiomics feature extraction process using IFoundry software (Intelligence Foundry 1.2, GE Healthcare), which considered 6940 radiomics features in six classes [53]. Similarly, Sun et al. [53] automatically extracted features using the Python package, Pyradiomics [61].

Zhao et al. [57] applied machine-learning-assisted feature extraction to filter predetermined statistical features based on their levels of importance (i.e., a feature reduction process) using the random forest algorithm. Additionally, Sun et al. [53] used the VGGNet-16 model [62] to serve as a deep feature extractor on elastographic images; notably, the team adopted a predetermined feature extraction approach on the B-mode images. Lastly, in six studies, researchers used a deep learning approach [48,49,50,51,52,54] in which the feature extraction and classification were nested and streamlined in an unsupervised manner.

### 4.3. Classification and Modeling

For traditional machine learning studies with separate feature extraction and classification processes, a broad spectrum of classifiers or statistical models have been explored, such as logistic regression, decision tree, naïve Bayes, etc. (Table 3). Notably, some researchers adopted a traditional machine learning approach (with separated feature extraction and classification processes) but used deep learning models as either deep feature extractors or classifiers, such as using convolutional neural network (CNN) as deep feature extractor and then connected to k-nearest neighbor (KNN) or extreme gradient boosting (XGBoost) that served as classifier.

As deep learning approaches, CNN and MLP were the typical methods considered. CNN receives input from image data, whereas MLP takes the flattened hue histogram matrix from the elastographic images [51,52]. Hu et al. [48] attempted to construct a series of CNN models using data from different segmentation settings. They applied a stochastic gradient descent of 0.9 momentum and 1 × 10^−4^ weight decays while assigning the cross-entropy loss as the loss function. The models were trained with a 128 batch size and 0.01 learning rate. Deep learning models were often pretrained using large datasets in the public domain, in which the ImageNet database [63] was commonly used. The pretraining process relieves the sample size demand in the actual training and can speed up convergence, especially during the early training stages [64].

Some compelling model architectures are worth discussing, particularly data fusion techniques in machine learning models. Sun et al. [53] separately trained the machine learning models on elastography and B-mode data and joined the two models by an uncertainty decision-theory-based voting system consisting of a pessimistic, optimistic, and compromise approach [65]. Pereira et al. [49] averaged the class probabilities of the B-mode- and elastography-data-trained models, which resembled the compromise approach of the voting system. Moreover, they applied a grid search approach on the weighted cross-entropy loss to determine the drop-out probability and learning rate.

Udriștoiu et al. [54] constructed a CNN and LSTM model using sequential ultrasound B-mode, elastographic, and Doppler images trained at 50 epochs. Then, they were merged by a concatenation layer. Qin et al. [50] investigated the differences in fusion methods, including mixed training fine-tuning, fusion followed by feature re-extraction, and feature extraction followed by refusion. In addition, they compared the fully connected layers, spatial pyramid pooling, and global average pooling for the classification layers [50].

To evaluate the model, in six studies, researchers divided the data into training and testing sets, whereas in one study, an external testing set was also used to improve generalizability [57]. In three studies, the authors adopted a cross-validation approach; in one study, both cross-validation and data-slicing approaches were implemented. In two studies, the validation method was not addressed.

### 4.4. Classification Performance

In the majority of the reviewed articles, the authors explored and compared the classification performance between different models or model architectures; Table 4 presents the best-performing or -featuring (in the abstract) model for each study. Accuracy and area under the receiver operating characteristics curve (AUC) were the primary outcomes and were presented in all but one article. AUC evaluates the model performance across different thresholds for a binary classifier, which represents the discriminatory power of a predictive model to distinguish between events and nonevents. Of the 10 articles reporting the accuracy measure, all methods attained an accuracy higher than 80% but only three exceeded 90% [50,54,57]. Methods for the pancreas appeared to be more accurate than those for the thyroid. The accuracy of methods in thyroid studies ranged from 83% to 94.7%, whereas that for methods in pancreas studies ranged from 84.27% to 98.26%. Additionally, all methods obtained a discriminatory power (via AUC) of more than 90%, except one.

Qin et al. [50] and Udriștoiu et al. [54] created the two models with the highest accuracy and discriminatory power: these models accounted for data fusion inside the deep learning model training process.

## 5. Discussion

In this contemporary review, we explored the applications of machine learning models of elastography for identifying tumors in the endocrine system. However, we only found mentions of ultrasound elastography in this review. Magnetic resonance elastography was available to facilitate the diagnosis of thyroid and pancreas cancer but might not be ready to incorporate with computer-aided diagnoses, such as in machine learning models [66,67,68]. In addition, only thyroid and pancreas tumors were captured by our review, which were assessed in B-mode with shear-wave ultrasound elastography and endoscopic ultrasound strain elastography, respectively. The difference was due to the organ location: the thyroid is superficially located. The use of elastography was not reported for other endocrine organs, such as the adrenal gland and pituitary, because they are not accessible or are beyond the detection depth of the elastography probe [69,70].

Traditional machine learning and deep learning models were common approaches in computer-aided diagnosis: in several studies, researchers combined different approaches to innovatively create unique model architectures, especially via data fusion. Ultrasound elastography often comes with B-mode ultrasound images with spatial information. Hu et al. [48] segmented elastography images by overlaying segmented B-mode images and compiling images with different segmentation approaches in a machine learning model. We also found different feature extraction strategies for B-mode and elastography images with a mixture of predetermined statistical or radiomics features and deep feature extractors. Pereira et al. [49] and Sun et al. [53] developed separate machine learning models for B-mode and elastography images and estimated the outcomes by averaging the probability output of the models. Moreover, Qin et al. [50] and Udriștoiu et al. [54] adopted a data fusion approach by integrating the data using mixed/sequential training and a concatenation layer, respectively, which yielded superior classification performance compared with the other methods in this review.

Reporting quality is an important attribute of publications, with studies of machine learning models being no exception [71]. Some reviewed articles did not present adequate details on the participants and protocols, which hinders the replication and interpretation of findings. Two of the eleven studies did not specify the ground truth reference of the diagnosis. Four studies did not present the demographic information of the patients. Five studies did not report the size of the tumor, which may affect the accuracy of segmentation. Furthermore, two studies reported neither the training–testing data division nor cross-validation of the model performance evaluation. The methodological quality of machine learning studies was also of particular concern, especially those with small sample sizes [72]. Some journals may target on the innovation of the modeling or architecture and may impose less stringent requirements on small dataset studies [73,74]. In this review, we found studies with dataset sizes of 60 to 70 subjects over 2 to 3 classes, which would be deemed insufficient. Data augmentation, transfer learning, and cross-validation are acceptable measures to accommodate the limitations of sample size and to handle over-fitting and convergence problems [50,73,75,76]. Additionally, imbalanced dataset classification is one of the pervasive challenges in machine learning. All studies in this review suffered from unbalanced class sizes, which might distort the validity of performance evaluation. Only one study accounted for the unbalanced class size using the bootstrapping approach [44]. Hyperparameters (or model parameters) are sets of parameters that must be configured for the model learning process [77]; the performance metrics of the model may be overly dependent on the tuning of hyperparameters [78]. The number of trees and nodes in the random forest classifier, the number of clusters (k) in the KNN models, and the number of layers in MLP models are typical model parameters. For deep learning, grid and random searches were common approaches to select the optimal combination of multiple hyperparameters, which are less time-consuming and require less computational resources [79]. We decided not to conduct a thematic and qualitative analysis on the selection of hyperparameters and optimization strategy (e.g., loss functions and cross-entropy), which deserves another standalone in-depth review in an engineering paper. However, three studies did not address the hyperparameter tuning strategies. Other studies mentioned their optimization strategies without the confirmed values of the hyperparameters or vice versa.

This review has some limitations. Due to language bias, some relevant research published in languages other than English could have been missed. Moreover, we only included journal articles indexed from the mentioned electronic databases, which we considered as higher quality but might constitute selection bias. In addition, we did not conduct a formal methodological quality assessment for the eligible articles because the focus of the studies was heterogeneous, which would have affected their efforts and focus on the direction of reporting. For example, some studies were more based on clinical applications, whereas others targeted on the innovations of the system development. Furthermore, our search results included articles with terms related to machine learning models. However, some boundaries between machine learning, advanced signal processing, and statistical techniques were ambiguous. Some studies may have been missed or their eligibility was difficult to determine, such as those using logistic regression [80].

Machine-learning-based ultrasound elastography is a recent technological advancement of the field because most of the articles were published after 2018. In addition to statistical models, progress can be observed in the direction of using deep learning models, mixed and sequential training, etc. Image processing or denoising plays an important role in the subsequent medical image analysis [81] but was less discussed in the reviewed studies. Machine learning or deep learning can also be applied in image denoising, segmentation, and augmentation. For example, generative adversarial network (GAN) was proven effective in semantic segmentation and generative image modeling for medical imaging [82,83], whereas linear combinations of datasets can be applied for data augmentation [84]. Additionally, we anticipate that integrating 3D B-mode ultrasound and 3D elastography will be the future trend in improving visualization and, thus, decision-making, as well as providing a complete profile of feature information.

Several core challenges are facing this field. Our review showed that the application of machine learning model technology remains at the initial stage. Despite most reviewed articles being contemporary, cutting-edge models were not used; researchers are still using non-deep-learning approaches. Features were mainly predetermined and relied on manual harvesting. Moreover, due to the size of the probe, penetration power, and constraints of shear-wave generation, elastography had not been applied for organs, such as adrenal and pituitary glands, as demonstrated in our review. Existing modalities also tend to reach the physical limits on resolution. Combining measurements with other physical properties may enhance our understanding of the features of tumors.

## 6. Conclusions

In this review, we summarized the applications and protocols of machine learning models on elastography to identify tumors in the endocrine system. Shear-wave ultrasound elastography has been applied to assess thyroid tumors, whereas strain elastography with endoscopy has been used for diagnosing pancreatic tumors. Machine learning approaches achieved an accuracy of >80%, whereas three studies reported an accuracy of >90%.

## Figures and Tables

**Table 1:** Subject and dataset information.

Reference	Sample Size	Sex (M:F)	Mean Age (years)	Type (B:M)	Size (mm)	Reference Standard
Hu et al. [48]	1582 patients 1747 nodules	567:1015	46.40 (SD: 9.65)	Thyroid 701:1046	14.51 ± 3.51	FNA
Pereira et al. [49]	165 patients 964 images	-	-	Thyroid 752:212	-	-
Qin et al. [50]	233 patients 1156 nodules	-	-	Thyroid 539:617	-	Verified by clinical pathology
Săftoiu et al. [51]	68 patients	47:21	Normal: 49.4 (SD: 15.4) ChPan: 55.1 (SD: 17.0) PanCA: 62.3 (SD: 12.9)	Normal: 22 ChPan: 11 PanCA: 35	-	CT and biopsy
Săftoiu et al. [52]	258 patients 774 recordings	172:76	PanCA: 64 (SD: 15.40) ChPan: 56 (SD: 13.25)	Pancreatic 47:211	PanCA: 31.97 (SD: 11.69, 6–85) ChPan: 28.36 (SD: 12.23, 9–60)	FNA biopsy, verified by clinical, biological exams, and repeated imaging tests
Sun et al. [53]	245 patients 490 images	-	-	Thyroid 145:100	-	Biopsy
Udriștoiu et al. [54]	65 patients 1300 images	-	-	PDAC: 30 CPP: 20 PNET: 15	-	FNA biopsy
Zhang et al. [55]	2032 patients 2064 nodules	695:1337	45.25 (SD: 13.49)	Thyroid 1314:750	≤25	-
Zhao et al. [56]	174 patients 177 nodules	45:132	B: 47.9 M: 41.9	Thyroid 81:96	B: 23.4 M: 20.0	FNA biopsy
Zhao et al. [57]	720 patients 743 nodules	168:552	49.61 (15–89)	Thyroid 469:274	≥10	Biopsy
Zhou et al. [58]	70 patients 107 nodes	10:60	30	Thyroid 32:75	≤10	FNA biopsy

B:M: benign-to-malignant ratio; ChPan: chronic pancreatitis; CPP: chronic pseudotumoral pancreatitis; FNA: fine-needle aspiration; M:F: male-to-female ratio; PanCA: pancreatic cancer; PDAC: pancreatic ductal adenocarcinoma; PNET: pancreatic neuroendocrine tumor; SD: standard deviation.

**Table 2:** Configuration of elastography system and image segmentation.

Articles	Mode	Type	System	Processing and Segmentation
Hu et al. [48]	US	SWE + B-mode	ACUSON Sequoia Redwood US diagnostic system (Siemens, Munich, Germany)	Use PP-LiteSeg to segment SWE by B-mode
Pereira et al. [49]	US	SWE + B-mode	-	Segmented SWE region with stress corresponding to 0.7 max stress value
Qin et al. [50]	US	SWE + B-mode	Aixplorer ultrasonic machine	Pre-extracted ROI by color channel transformation and segmented by radiologists
Săftoiu et al. [51]	US	EUS, SE + B-mode	HITACHI 8500 (Hitachi Medical Systems, Zug, Switzerland) Pentax Linear Endoscope EG3830UT and EG3870 UTK (Pentax, Hamburg Germany)	Processed using ImageJ software to extract hue histogram matrix. Manual selection or tumor area
Săftoiu et al. [52]	US	EUS	-	Processed using a special software plugin based on ImageJ software to extract hue histogram matrix. Manual selection or tumor area.
Sun et al. [53]	US	SWE + B-mode	-	ROI manually segmented using ITK-SNAP Denoise with Median Filter and outlined by radiologists.
Udriștoiu et al. [54]	US	EUS, SE, Doppler	HITACHI Preirus EG3870UTK, Pentax Medical Corporation	Contrast enhancement, ROI manual segmentation
Zhang et al. [55]	US	SE + B-mode	HI Vision 900, HI Vision Ascendus, HI Vision Preirus color US units from Hitachi (Tokyo, Japan)	Conducted by experienced radiologist
Zhao et al. [56]	US	SE + B-mode	HITACHI Vision 900 system (Hitachi Medical System, Tokyo, Japan), -	-
		B-mode	Philips iu222 (Philips, Bothell, WA, USA)	
Zhao et al. [57]	US	SWE + B-mode	Aixplorer; Supersonic Imagine (Paris, France), SWE	ROI extracted by Q-Box quantification tool
Zhou et al. [58]	US	-	-	Contrast enhancement

EUS: endoscopic ultrasound; ROI: region of interest; SE: strain elastography; SWE: shear-wave elastography; US: ultrasound.

**Table 3:** Configuration of machine learning and classification models.

Articles	Feature Extraction Strategy	Classifier/Model	Validation (trn:tst)
Hu et al. [48]	7 ResNet18 models on different segmentation approaches		71:29
Pereira et al. [49]	Predetermined SWE statistical features and SWE features extracted by circular Hough transform	Logistic regression, naïve Bayes, SVM, decision tree	82:18
	Fully trained CNN (2-layer) model for B-mode and SWE Pretrained CNN18 for B-mode and SWE Combine classification by averaging class probabilities of trained B-mode and SWE models		
Qin et al. [50]	Pretrained VGG16 with 3 fused methods (MT, FEx-reFus, and Fus-reFEx) and 3 classifier layers (FCL, SPP, and GAP)		82:18
Săftoiu et al. [51]	MLP (3- and 4-layer)		10-fold cxv
Săftoiu et al. [52]	MLP (4-layer)		10-fold cxv
Sun et al. [53]	Deep feature extractor on SWE US Predetermined statistical and radiomics features on B-mode US	Logistic regression, naïve Bayes, and SVM on both SWE and B-mode features. Classifications of both models were combined and hybridized by uncertainty decision-theory-based voting system (pessimistic, optimistic, and compromise approaches).	5-fold cxv
Udriștoiu et al. [54]	CNN on B-mode, contrast harmonic sequential images taken at 0, 10, 20, 30, 40 s, color Doppler, and elastography LSTM on contrast harmonic sequential images taken at 0, 10, 20, 30, 40 s CNN and LSTM merged by concatenation layer.		80:20
Zhang et al. [55]	11 predetermined B-mode features 1 predetermined elastography feature	Logistic regression, linear discriminant analysis, random forest, kernel SVM, adaptive boosting, KNN, neural network, naïve Bayes, CNN	60:40, 10-fold cxv
Zhao et al. [56]	20 predetermined radiomics features	Logistic regression, random forest, XGBoost, SVM, MLP, KNN	-
Zhao et al. [57]	Machine-learning-assisted approach (6 predetermined B-mode and 5 SWE features) Radiomics features	Decision tree, naïve Bayes, KNN, logistic regression, SVM, KNN-based bagging, random forest, XGBoost, MLP, gradient boosting tree	Training: 520 Testing: 223 External Testing: 106
Zhou et al. [58]	Predetermined statistical features, Feature extraction by GLCOM-GLRLM, MSCOM	RBM + Bayesian	-

CNN: convolutional neural network; FCL: fully connected layers; FEx-reFus: feature extraction followed by refusion; Fus-reFEx: fusion followed by feature re-extraction; GAP: global average pooling; GLCOM-GLRLM: ray-level co-occurrence matrix and gray-level run-length matrix; KNN: k-nearest neighborhood; LSTM: long short-term memory; MLP: multilayer perceptron; MSCOM: multiple subgraph co-occurrence matrix based on multilevel wavelet; MT: mixed training; RBM: restricted Boltzmann machine; SPP: spatial pyramid pooling; SVM: support vector machine; SWE: shear-wave elastography; trn: training; tst; testing; cxv: cross-validation; XGBoost: extreme gradient boosting.

**Table 4:** Evaluation metric and outcome performance of the method with either the best or featured model.

Articles	Model and Approach	Evaluation Metrics and Outcomes					
		Acc (%)	Sn/Rc (%)	Sp (%)	PPV/Pc (%)	NPV (%)	AUC (%)
Hu et al. [44]	B-mode + SWE (1.0 mm offset) ResNet18	86.45	85.15	91.93	82.12	73.54	93
Pereira et al. [45]	SWE Pretrained CNN18	83	-	-	-	-	80
Qin et al. [46]	Pretrained VGG16 Ex-reFus with SPP	94.7	92.77	**97.96**	-	-	**98.77**
Săftoiu et al. [47]	MLP (3-layer)	89.7	91.4	87.9	88.9	90.6	95
Săftoiu et al. [48]	MLP (2-layer)	84.27	87.59	82.94	96.25	57.22	94
Sun et al. [49]	Hybridized model with voting system (compromise approach)	86.5	82	89.7	-	-	92.1
Udriștoiu et al. [50]	CNN-LSTM	**98.26**	**98.6**	97.4	**98.7**	**97.4**	98
Zhang et al. [51]	Random forest	85.7	89.1	85.3	-	-	93.8
Zhao et al. [52] 2020	Random forest	86.0	86.6	85.5	-	-	93.4
Zhao et al. [53] 2021	Machine-learning-assisted approach (B-mode + SWE) using KNN-based bagging model	93.4	93.9	93.2	86.1	97.1	95.3
Zhou et al. [54]	RBM + Bayesian (UE)	-	90.21	78.45	-	-	-

Acc: accuracy; AUC: area under receiver operator characteristic curve; CNN: convolutional neural network; KNN: k-nearest neighbor; LSTM: long short-term memory; MLP: multilayer perceptron; NPV: negative predictive value; Pc: precision; PPV: positive predictive value; RBM: restricted Boltzmann machine; Rc: recall; Sn: sensitivity; Sp: specificity; SWE: shear-wave elastography. Bold typeface indicates the best performance among the methods.

## Data Availability

The data can be shared up on request.

## Supplementary Materials

The following supporting information can be downloaded at: https://www.mdpi.com/article/10.3390/cancers15030837/s1, Table S1: Search terms and operations.
