# Supplementary material for: Endocrine Tumor Classification via Machine-Learning-Based Elastography: A Systematic Scoping Review
Source: Cancers (Basel). 2023 Jan 29;15(3):837. doi: 10.3390/cancers15030837 (PMC9913672; doi:10.3390/cancers15030837)
Supplement: Supplementary file 1 [file cancers-15-00837-s001.zip › cancers-2096259-supplementary.pdf]

Table S1. Search terms and operations.

Database	Search Field	Search Terms
SCOPUS	Title/Abstract/Keywords	(thyroid OR pancrea* OR adrenal OR endocrine) AND (elastograph* OR sonoelastograph*) AND (nodule* OR tumor OR tumour OR cancer OR carcinoma OR malignan* OR neoplas* OR mass*) AND ((machine learning) OR (deep learning) OR (neural network) OR CNN OR RNN OR ANN OR (cascaded networks) OR (deep cascade learning) OR GAN OR GANs)
Web of Science	topic (Title/Abstract/Author keywords/Keywords plus)	(thyroid OR pancrea* OR adrenal OR endocrine) AND (elastograph* OR sonoelastograph*) AND (nodule* OR tumor OR tumour OR cancer OR carcinoma OR malignan* OR neoplas* OR mass*) AND ((machine learning) OR (deep learning) OR (neural network) OR CNN OR RNN OR ANN OR (cascaded networks) OR (deep cascade learning) OR GAN OR GANs)
IEEEXpress	Metadata (Title/Abstract/Indexing terms)	(thyroid OR pancrea* OR adrenal OR endocrine) AND (elastograph* OR sonoelastograph*) AND (nodule* OR tumor OR tumour OR cancer OR carcinoma OR malignan* OR neoplas* OR mass*) AND (machine learning OR deep learning OR neural network OR CNN OR RNN OR ANN OR cascaded networks OR deep cascade learning OR GAN OR GANs)
PubMed	Title/Abstract	(thyroid OR pancrea* OR adrenal OR endocrine) AND (elastograph* OR sonoelastograph*) AND (nodule* OR tumor OR tumour OR cancer OR carcinoma OR malignan* OR neoplas* OR mass*) AND ((machine learning) OR (deep learning) OR (neural network) OR CNN OR RNN OR ANN OR (cascaded networks) OR (deep cascade learning) OR GAN OR GANs)
EMBASE	Title/Abstract/Author keywords	(thyroid OR pancrea* OR adrenal OR endocrine) AND (elastograph* OR sonoelastograph*) AND (nodule* OR tumor OR tumour OR cancer OR carcinoma OR malignan* OR neoplas* OR mass*) AND ((machine learning) OR (deep learning) OR (neural network) OR CNN OR RNN OR ANN OR (cascaded networks) OR (deep cascade learning) OR GAN OR GANs)
CINAHL (via EbscoHost)	Default (Title/Abstract/Keywords)	(thyroid OR pancrea* OR adrenal OR endocrine) AND (elastograph* OR sonoelastograph*) AND (nodule* OR tumor OR tumour OR cancer OR carcinoma OR malignan* OR neoplas* OR mass*) AND ((machine learning) OR (deep learning) OR (neural network) OR CNN OR RNN OR ANN OR (cascaded networks) OR (deep cascade learning) OR GAN OR GANs)
